# Supplementary material for: Quality of Life vs. Supportive Care Needs for Oral Cancer Caregivers: Are They Related?
Source: Curr Oncol. 2023 Feb 1;30(2):1733–44. doi: 10.3390/curroncol30020134 (PMC9954937; doi:10.3390/curroncol30020134)
Supplement: Supplementary file 1 [file curroncol-30-00134-s001.zip › File S1_M-CNAT-C.pdf]

Penilaian Komprehensif Keperluan Penjaga Pesakit Kanser (CNAT-C)
Comprehensive Needs Assessment Tool for Cancer Caregiver (CNAT-C)

Sila baca setiap pernyataan dengan teliti dan jawab berdasarkan pengalaman anda pada bulan yang lalu.
Please read each statement carefully and answer based on your experience over the past month.

Untuk setiap pernyataan, sila tanda (√) pada kotak yang sesuai.
For each statement, please tick (√) the appropriate box.

Sekiranya anda memerlukan bantuan mengenai pernyataan berikut, sila tandakan isikan (√) di dalam kotak yang paling sesuai dengan tahap keperluan anda:
1 = memerlukan bantuan yang sedikit
2 = memerlukan bantuan secara sederhana
3 = memerlukan bantuan yang banyak
di bawah bahagian "Perlu bantuan Keperluan (Y)".

If you need help with the following statements, please tick (√) the box that best suits your needs:
1 = need a little help
2 = need moderate help
3 = need a lot of help
under the "Need Help (O)" section.

Sebagai contoh, pernyataan "Saya memerlukan maklumat mengenai ujian dan rawatan" merujuk kepada pengalaman anda pada bulan lalu untuk mendapatkan maklumat mengenai ujian dan rawatan.
For example, the statement "I need information about tests and treatments" refers to your experience over the past month to get information about tests and treatments.

Sekiranya anda merasakan yang anda menghadapi kesukaran untuk mendapatkan maklumat mengenai ujian dan rawatan pada bulan lalu dan merasa bahawa anda memerlukan bantuan secara sederhana, sila tanda pada kotak "Sederhana" di bahagian "Perlu bantuan Keperluan (Y)" seperti contoh di bawah.
If you feel you are having difficulty getting information about tests and treatments in the past month and feel that you need moderate help, please tick (√) the "Moderate" box in the "Need Help (O)" section as in the example below:

**Pada bulan lalu**<br>***In the past month***	**Saya tidak perlu bantuan (X)**<br>***No need help (X)***	**Perlu bantuan (Y)**<br>***Need help (Y)***		
		**Sedikit**<br>***A little***	**Sederhana**<br>***Moderate***	**Banyak**<br>***A lot***
	**(0)**	**(1)**	**(2)**	**(3)**
Saya memerlukan maklumat mengenai ujian dan rawatan<br>*I need information about tests and treatments*			✓	

Sila tanda (√) pada kotak 'Tidak perlu bantuan (X)' dalam kes-kes berikut:
Please tick (√) the 'No need help (X)' box in the following cases:

① Ia adalah tidak berkenaan dengan anda ATAU
It is not related to you OR

② Anda tidak menghadapi sebarang masalah ATAU
You do not face any problems OR

③ Anda menghadapi masalah, namun merasakan bahawa anda tidak memerlukan bantuan, atau tidak mahu menerima bantuan.
You have a problem, but feel that you do not need help, or refuse to receive help.

**Pada bulan lalu** *In the past month*	**Saya tidak perlu bantuan (X)** *No need help (X)*	**Perlu bantuan (Y)** *Need help (Y)*		
		**Sedikit** *A little*	**Sederhana**	**Banyak**
	**(0)**	**(1)**	**(2)**	**(0)**
Saya memerlukan maklumat mengenai ujian dan rawatan *I need information about tests and treatments*	✓			

No. *No.*	Pada bulan lalu *In the past month*	Saya tidak perlu bantuan (X) *No need help (X)*	Perlu bantuan (Y) *Need help (Y)*		
			Sedikit *A little*	Sederhana	Banyak
		(0)	(1)	(2)	(0)
1.	Saya memerlukan bantuan berkaitan masalah kesihatan saya. *I needed help with my health problems.*				
2.	Saya memerlukan bantuan dengan perkara berkaitan pesakit. *I needed help with concerns about the patient.*				
3.	Saya memerlukan bantuan untuk menangani kemurungan. *I needed help to deal with depression.*				
4.	Saya memerlukan bantuan untuk menangani rasa marah, mudah marah (jengkel), atau gementar. *I needed help to deal with feelings of anger, irritability, or nervousness.*				
5.	Saya memerlukan bantuan untuk menangani kesunyian atau rasa terasing / tersisih. *I needed help to deal with loneliness or feelings of isolation.*				
6.	Saya memerlukan bantuan untuk menangani rasa gelisah yang tidak ketara. *I needed help to deal with indefinite anxiety.*				

No.<br>*No.*	Pada bulan lalu<br>*In the past month*	Saya tidak perlu bantuan (X)<br>*No need help (X)*	Perlu bantuan (Y)<br>*Need help (Y)*		
			Sedikit<br>*A little*	Sederhana	Banyak
		(0)	(1)	(2)	(0)
7.	Saya memerlukan bantuan untuk menghadapi pesakit yang terlampau bergantung.<br>*I needed help dealing with overly dependent patients.*				
8.	Saya memerlukan bantuan untuk menghadapi pesakit yang kurang menghargai penjagaan yang saya berikan.<br>*I needed help dealing with patient's lack of appreciation about my caregiving.*				
9.	Saya memerlukan bantuan untuk menghadapi masalah / ketegangan dalam hubungan keluarga selepas diagnosis kanser.<br>*I needed help dealing with problems / tensions in family relationships after the cancer diagnosis.*				
10.	Saya memerlukan bantuan untuk menghadapi masalah dalam hubungan saya dengan orang lain (kawan, rakan sekerja, jiran dll.) selepas diagnosis kanser.<br>*I needed help dealing with problems in my relationships with others (friends, co-workers, neighbours etc.) after the cancer diagnosis.*				
11.	Saya memerlukan bantuan untuk relaks dan dalam kehidupan peribadi saya.<br>*I needed help to relax and in my personal life.*				
12.	Saya memerlukan doktor menunjukkan minat dan empati terhadap pesakit dan keluarga.<br>*I needed the doctor to show interest and empathy towards the patient and family members.*				
13.	Saya memerlukan doktor memberi penerangan yang jelas, spesifik, dan jujur.<br>*I needed the doctor to give a clear, specific, and honest explanation.*				
14.	Saya perlu berjumpa doktor dengan cepat dan mudah apabila perlu.<br>*I needed to see the doctor quickly and easily when necessary.*				

No.<br>*No.*	Pada bulan lalu<br>*In the past month*	Saya tidak perlu bantuan (X)<br>*No need help (X)*	Perlu bantuan (Y)<br>*Need help (Y)*		
			Sedikit<br>*A little*	Sederhana	Banyak
		(0)	(1)	(2)	(0)
15.	Saya memerlukan pesakit dan keluarga untuk turut terlibat dalam proses membuat keputusan dalam memilih sebarang ujian atau rawatan yang pesakit terima.<br>*I needed the patient and family members to be involved in the decision-making process in choosing any tests or treatments that patients receive.*				
16.	Saya memerlukan kerjasama dan komunikasi yang baik di kalangan kakitangan penjagaan kesihatan.<br>*I needed good cooperation and communication among health personnel.*				
17.	Saya memerlukan jururawat menunjukkan minat dan empati terhadap pesakit dan keluarga.<br>*I needed the nurse to show interest and empathy towards the patient and family members.*				
18.	Saya memerlukan jururawat menerangkan rawatan atau penjagaan yang diberikan kepada pesakit.<br>*I needed the nurse to explain the treatment or care given to the patient.*				
19.	Saya memerlukan jururawat segera mengatasi ketidakselesaan dan kesakitan pesakit.<br>*I needed the nurse to immediately address the patient's discomfort and pain.*				
20.	Saya memerlukan maklumat berkenaan status terkini pesakit dan prognosis (ramalan kesembuhan) penyakit.<br>*I needed information on the patient's current status and the prognosis (future course) of the disease.*				
21.	Saya memerlukan maklumat mengenai ujian dan rawatan yang diterima oleh pesakit.<br>*I needed information on tests and treatments received by the patient.*				

No.<br>*No.*	Pada bulan lalu<br>*In the past month*	Saya tidak perlu bantuan (X)<br>*No need help (X)*	Perlu bantuan (Y)<br>*Need help (Y)*		
			Sedikit<br>*A little*	Sederhana	Banyak
		(0)	(1)	(2)	(0)
22.	Saya memerlukan maklumat mengenai cara penjagaan pesakit (menangani tanda-tanda penyakit, diet, senaman dll.).<br>*I needed information on how to care for the patient (dealing with symptoms of illness, diet, exercise etc.).*				
23.	Saya memerlukan panduan atau maklumat mengenai perubatan komplimentari dan alternatif.<br>*I needed guidelines or information on complementary and alternative medicine.*				
24.	Saya memerlukan maklumat mengenai hospital atau klinik dan doktor yang merawat kanser.<br>*I needed information about the hospitals or clinics and doctors treating cancer.*				
25.	Saya memerlukan maklumat mengenai bantuan kewangan untuk perbelanjaan perubatan sama ada daripada organisasi kerajaan dan / atau swasta.<br>*I needed information on financial support for medical expenses either from the government and / or private organizations.*				
26.	Saya memerlukan bantuan dalam komunikasi dengan pesakit dan / atau ahli keluarga yang lain.<br>*I needed help in communicating with the patient and / or other family members.*				
27.	Saya memerlukan maklumat mengenai pengurusan stres berkaitan penjagaan pesakit.<br>*I needed information on stress management related to patient caregiving.*				
28.	Saya memerlukan sokongan agama.<br>*I needed religious support.*				
29.	Saya memerlukan bantuan dalam mencari makna / hikmah situasi saya dan menerimanya.<br>*I needed help in finding the meaning / reason of my situation and accepting it.*				

No.<br>*No.*	Pada bulan lalu<br>*In the past month*	Saya tidak perlu bantuan (X)<br>*No need help (X)*	Perlu bantuan (Y)<br>*Need help (Y)*		
			Sedikit<br>*A little*	Sederhana	Banyak
		(0)	(1)	(2)	(0)
30.	Saya memerlukan seorang kakitangan hospital yang khusus untuk memberi kaunseling dan bimbingan sepanjang tempoh rawatan sehingga selepas discaj.<br>*I needed a specific hospital staff to provide counseling and guidance throughout the treatment period until after discharge.*				
31.	Saya memerlukan panduan mengenai kemudahan dan perkhidmatan yang disediakan oleh hospital.<br>*I needed guidance on the facilities and services provided by the hospital.*				
32.	Saya memerlukan ruang khusus untuk penjaga pesakit di hospital.<br>*I needed an allocated space for the patient's caregiver in the hospital.*				
33.	Saya memerlukan jururawat memberikan perkhidmatan di rumah.<br>*I needed a nurse to provide services at home.*				
34.	Saya memerlukan peluang untuk berkongsi pengalaman atau maklumat dengan penjaga lain.<br>*I needed an opportunity to share experience or information with other caregivers.*				
35.	Saya memerlukan perkhidmatan kebajikan (seperti kaunseling) untuk penjaga pesakit.<br>*I needed welfare services (such as counselling) for patient's caregivers.*				
36.	Saya memerlukan perkhidmatan pengangkutan untuk berulang-alik ke hospital.<br>*I needed transportation service to get to and from the hospital.*				
37.	Saya memerlukan tempat rawatan yang berdekatan dengan rumah pesakit.<br>*I needed a nearby treatment place to the patient's home.*				

No.<br>*No.*	Pada bulan lalu<br>*In the past month*	Saya tidak perlu bantuan (X)<br>*No need help (X)*	Perlu bantuan (Y)<br>*Need help (Y)*		
			Sedikit<br>*A little*	Sederhana	Banyak
		(0)	(1)	(2)	(0)
38.	Saya memerlukan penginapan berhampiran dengan hospital di mana pesakit dirawat.<br>*I needed accommodation near the hospital where the patient is treated.*				
39.	Saya memerlukan bantuan untuk menangani beban ekonomi yang disebabkan oleh kanser.<br>*I needed help to deal with the economic burden caused by cancer.*				
40.	Saya memerlukan bantuan seseorang untuk menguruskan rumahtangga dan / atau menjaga anak.<br>*I needed someone to help manage the household and / or child care.*				
41.	Saya memerlukan bantuan dalam penjagaan pesakit di hospital atau di rumah.<br>*I needed help with caring for the patient in the hospital or at home.*				

Adakah anda menghadapi masalah lain yang tidak disenaraikan di atas dan memerlukan bantuan? (Sila jelaskan)
Are you having other problems not listed above and need help? (Please explain)

____

____
