# Supplementary material for: Quality of Life vs. Supportive Care Needs for Oral Cancer Caregivers: Are They Related?
Source: Curr Oncol. 2023 Feb 1;30(2):1733–44. doi: 10.3390/curroncol30020134 (PMC9954937; doi:10.3390/curroncol30020134)
Supplement: Supplementary file 1 [file curroncol-30-00134-s001.zip › File S2_M-CarGOQoL.pdf]

Soal Selidik Kualiti Hidup Penjaga Onkologi (CarGOQoL)
Caregiver Oncology Quality of Life Questionnaire (CarGOQoL)

Sila jawab setiap soalan dengan menandakan jawapan yang paling hampir dengan apa yang anda fikirkan atau rasakan selama **empat minggu yang lalu**. Beberapa soalan adalah berkaitan dengan kehidupan peribadi anda. Soalan-soalan ini adalah perlu untuk menilai setiap aspek kualiti kehidupan anda. Walau bagaimanapun, jika anda tidak tahu bagaimana untuk menjawab, atau jika soalan tersebut tidak berkaitan, anda boleh menjawab soalan seterusnya.

Please answer each question by ticking the answer that is closest to what you thought of felt ***during the past four weeks****. Some of the questions are related to your personal life. These questions are necessary to evaluate every aspect of your quality of life. However, if you are not sure on how to answer the questions, or the questions are not relevant (not related to you), you can skip to the next question.*

No.<br>***No.***	Selama empat minggu yang lalu, berkaitan dengan pesakit yang anda bantu, pernahkah anda…<br>*During the past four weeks, in relation to the person you help, have you…*	Tidak pernah / Tidak sama sekali<br>*Never / Not at all*	Jarang / Sedikit<br>*Rarely / Few*	Kadang-kadang / Sederhana<br>*Sometimes / Moderately*	Kerap / Banyak<br>*Often / Mostly*	Selalu / Sangat<br>*Always*
1.	Merasa bimbang, cemas?<br>*Felt worried, anxious?*					
2.	Merasa sedih, murung?<br>*Felt sad, depressed?*					
3.	Merasa letih, penat dari segi emosi?<br>*Felt tired, exhausted emotionally?*					
4.	Merasa tertekan?<br>*Felt stressed?*					
5.	Merasa kebebasan anda terhalang?<br>*Felt your freedom is restricted?*					
6.	Merasa terganggu dengan perasaan seperti terkurung?<br>*Felt disturbed by the feeling of being confined?*					
7.	Merasa terganggu dengan kenyataan bahawa hidup anda adalah tertumpu sepenuhnya untuk menjaga pesakit?<br>*Felt disturbed by the fact that your life is completely focused on caring for the patient?*					
8.	Merasa terganggu menjadi satu-satunya orang yang memberikan bantuan?<br>*Felt disturbed that you are the only person to provide help?*					

No.<br><br><br>***No.***	Selama empat minggu yang lalu, berkaitan dengan pesakit yang anda bantu, pernahkah anda…<br>*During the past four weeks, in relation to the person you help, have you…*	Tidak pernah / Tidak sama sekali<br><br>*Never / Not at all*	Jarang / Sedikit<br><br>*Rarely / Few*	Kadang-kadang / Sederhana<br><br>*Sometimes / Moderately*	Kerap / Banyak<br><br>*Often / Mostly*	Selalu / Sangat<br><br>*Always*
9.	Berpuas hati dengan maklumat yang diberikan oleh kakitangan kesihatan (doktor, jururawat)?<br>*Felt satisfied with the information provided by health personnel (doctors, nurses..)?*					
10.	Diyakinkan oleh kakitangan kesihatan (doktor, jururawat) mengenai peranan anda sebagai penjaga?<br>*Felt reassured by health personnel (doctors, nurses) about your role as a caregiver?*					
11.	Merasa bahawa peranan anda sebagai penjaga diakui oleh kakitangan kesihatan (doktor, jururawat)?<br>*Felt that your role as a caregiver is recognized by health personnel (doctors, nurses…)?*					
12.	Menghadapi masalah kewangan (berkaitan tempat tinggal, pengangkutan dll.)?<br>*Had financial problems (related to accommodation, transportation etc.)?*					
13.	Menghadapi masalah lain (berkaitan tempat tinggal, pengangkutan dll.)?<br>*Had other problems (related to accommodation, transportation etc.)?*					
14.	Menghadapi masalah urusan dokumentasi (kertas kerja berkenaan insurans kesihatan jika ada, dan dokumen lain yang berkaitan dengan kanser)?<br>*Had problems with documentation (paperwork on health insurance if any, and other documents related to cancer)?*					

No.<br><br><br><br>***No.***	Selama empat minggu yang lalu, berkaitan dengan pesakit yang anda bantu, pernahkah anda…<br>*During the past four weeks, in relation to the person you help, have you…*	Tidak pernah / Tidak sama sekali<br><br>*Never / Not at all*	Jarang / Sedikit<br><br>*Rarely / Few*	Kadang-kadang / Sederhana<br><br>*Sometimes / Moderately*	Kerap / Banyak<br><br>*Often / Mostly*	Selalu / Sangat<br><br>*Always*
15.	Merasa bersalah?<br>*Felt guilty?*					
16.	Merasa terganggu dengan perasaan tidak berdaya untuk berbuat apa-apa berkenaan penyakit ini?<br>*Felt disturbed by the feeling of not being able to do anything about the disease?*					
17.	Merasa tidak adil, marah atau memberontak?<br>*Felt a feeling of unfairness, anger or rebellion?*					
18.	Menghadapi masalah tidur?<br>*Had trouble sleeping?*					
19.	Menghadapi masalah kurang selera makan?<br>*Had lack of appetite?*					
20.	Merasa letih, penat dari segi fizikal?<br>*Felt tired, exhausted physically?*					
21.	Beranggapan bahawa kesihatan anda lemah?<br>*Had the impression that your health was weak?*					
22.	Merasa bahawa anda telah membawa perubahan kepada orang yang anda bantu?<br>*Felt that you made a difference to the person you helped?*					
23.	Rasa berguna?<br>*Felt useful?*					
24.	Dapat berehat, relaks?<br>*Been able to rest and relax?*					
25.	Dapat menjaga diri, dan memberi perhatian terhadap kesihatan diri sendiri?<br>*Been able to take care of yourself, and pay attention to your own health?*					

No.<br><br><br>***No.***	Selama empat minggu yang lalu, berkaitan dengan pesakit yang anda bantu, pernahkah anda…<br>*During the past four weeks, in relation to the person you help, have you…*	Tidak pernah / Tidak sama sekali<br><br>*Never / Not at all*	Jarang / Sedikit<br><br>*Rarely / Few*	Kadang-kadang / Sederhana<br><br>*Sometimes / Moderately*	Kerap / Banyak<br><br>*Often / Mostly*	Selalu / Sangat<br><br>*Always*
26.	Dibantu, disokong, difahami oleh keluarga anda?<br>*Been assisted, supported and understood by your family?*					
27.	Dibantu, disokong, difahami oleh kawan-kawan anda?<br>*Been assisted, supported and understood by your friends?*					
28.	Menghadapi masalah dalam kehidupan intim dan emosi anda?<br>*Had problems in your intimate and emotional life?*					
29.	Mempunyai hubungan cinta dan seks yang memuaskan?<br>*Had a satisfying love and sex relationship?*					
